# Group theoretical analysis for unsteady magnetohydrodynamics flow and radiative heat transfer of power-law nanofluid subject to Navier’s slip conditions

**DOI:** 10.1371/journal.pone.0258107

**Published:** 2021-10-08

**Authors:** Saba Javaid, Asim Aziz, Taha Aziz

**Affiliations:** 1 Department of Mathematics, School of Natural Sciences, National University of Sciences and Technology, Islamabad, Pakistan; 2 College of Electrical and Mechanical Engineering, National University of Sciences and Technology, Rawalpindi, Pakistan; 3 Department of Mathematics, Dammam Community College, King Fahd University of Petroleum and Minerals, Dhahran, Saudi Arabia; 4 Interdisciplinary Research Center for Hydrogen and Energy Storage, King Fahd University of Petroleum and Minerals, Dhahran, Saudi Arabia; Tongji University, CHINA

## Abstract

The present work covers the flow and heat transfer model for the Power-law nanofluid in the presence of a porous medium over a penetrable plate. The flow is caused by the impulsive movement of the plate embedded in Darcy’s porous medium. The flow and heat transfer models are examined with the effect of linear thermal radiation in the flow regime. The Rosseland approximation is utilized for the optically thick nanofluid. The governing partial differential equations are solved using Lie symmetry analysis to find the reductions and invariants for the closed-form solutions. These invariants are then utilized to obtain the exact solutions for the shear-thinning, Newtonian, and shear-thickening nanofluids. In the end, all solutions are plotted for the *Cu*-water nanofluid to observe the effect of different emerging flow and heat transfer parameters.

## Introduction

Dispersion of nanoparticles in the ordinary heat transfer fluids presents new heat transfer innovations. These so called *nanofluids* enables the production and operations of smaller and lighter cooling systems for use in micro-electro-mechanical systems (MEMS) and nano-electro-mechanical systems (NEMS). Nanofluids also have applications in fields like, targeted drug delivery in patients during emergency treatment, manufacturing of energy efficient thermal solar system, cooling of microprocessors, nuclear systems, smart glass manufacturing, oil extraction, hydraulic braking systems [1–9]. In all mentioned processes, the ability to control the transfer of heat is important because the final quality of the product depends on this. Nanoparticles must be stable and are of appropriate shape and size to avoid clogging and sedimentation within the heat transfer equipment. A wide range of applications of nanofluids in thermal systems led researchers to study nanofluid flow and heat transfer processes under different thermophysical conditions and geometries. Ferdows *et al*. [10] studied the behaviour of viscous nanofluid in the presence of internal heat generation and suction by considering copper and aluminium nanoparticles. The governing equations are solved numerically after utilizing the similarity transformations to see the behaviour of key parameters of flow and heat transfer. The main findings of the research include the enhancement in the temperature with augmentation in the Eckert number and reduction with rising values of radiation term. The study of two dimensional stagnation point flow of nanofluid with heat transfer affected by solar radiation is reported by Ghasemi and Hatami [11]. The numerical results presented here establish that the temperature and thermal boundary layer thickness are increasing functions of Biot number. The survey articles of Eastman [12], Wang *et al*. [13], Keblinski *et al*. [14], Buongiorno [15], Das *et al*. [16], Vanaki *et al*. [17], Zhang *et al*. [18] and Okonkwo *et al*. [19] and the references therein provide comprehensive literature on the flow of nanofluids over the flat surface and characteristics of heat transfer processes.

Nanofluids are often believed to be Newtonian fluids in available studies. However, recently the researchers are using non-Newtonian fluid models for analysis of nanofluids. This is in line with the overall molecular behaviour/characteristics of nanofluids. The power-law model is an easy-to-use model suitable for shear-thickening and shear-thinning fluids. If fluid viscosity increases as the applied stress increase it is called the dilatant or shear-thickening fluid. Pseudo-plastics or shear-thinning fluids are defined by the opposite relationship between the viscosity and the applied stress. Santra *et al*. [20] examined heat transfer in *Cu*-water nanofluid when fluid is moving through a rectangular channel. They considered the power-law model for the laminar non-Newtonian nanofluid and numerical solutions are approximated using the finite volume method. Their results show an increase in the rate of heat transfer because of the presence of nanofluids. Hojjat *et al*. [21] set up an experiment that includes uniform distribution of nanoparticles in the base fluid. After the disbursement of nanoparticles, the shear-thinning behaviour for nanofluid is observed. It is also observed that the rheological properties of nanofluid differ with temperature variations and the concentration of nanoparticles. Khan and Golra [22] presented similar solutions for the temperature distribution and mass transfer of power-law nanofluid over a moving surface. This work is extended by the Khan and Khan [23] for the case of MHD boundary layer flow of power-law nanofluid. They have concluded that the velocity and temperature profiles are greatly affected by variations in a power-law index. Aziz and Jamshed [24] later included the slip and varying thermal conductivity effect in the model. Recently, Deng [25] and Deng *et al*. [26] presented the thermal behaviour of power-law nanofluid in a rectangular microchannel. It is concluded that the heat transfer rate of electro-osmotic flow is enhanced for shear thickening nanofluids. The readers are also recommended to study the latest research on power-law nanofluids [27–34].

The literature survey reveals that the mathematical models involving non-Newtonian nanofluids are mostly solved using numerical and experimental techniques. The nonlinear nature of modelled differential equations makes them difficult to solve analytically. The exact (closed-form) solutions are often preferred over numerical or experimental results to study the physical behaviour of the fluid flow. Moreover, these are also used to determine the reliability of both computational and iterative approaches. There are few studies available in which exact solutions are sought for the nonlinear models. Maghsoudi *et al*. [35] using the Galerkin process studied the MHD flow and radiative heat transfer characteristics of non-Newtonian nanofluid. The nanofluid is assumed to flow between two infinite vertical flat plates. The study showed that by increasing the magnetic force, the thermal efficiency decreases and there is a decreasing effect of the radiation parameter on the skin friction coefficient and the Nusselt number. Lie symmetry method is used to classify the closed-form solutions for the MHD flow and heat transfer of third-grade nanofluid with thermal radiation [36]. The same method is used by Aziz *et al*. [37] to find the solutions for MHD Stokes’ flow and radiative heat transfer of third-grade nanofluid with constant heat generation/absorption factor. The exact solution of the Stokes’ flow of a non-Newtonian nanofluid in a porous medium by considering the Navier’s slip condition is studied in [38]. Tahiri and Mansouri [39] form analytical solutions for the flow of non-Newtonian nanofluid inside a circular tube by applying the Laplace-Ritz variational method. The authors discussed the effect of different emerging parameters on the velocity and temperature of nanofluid. Kezzar *et al*. [40] presented the model of natural convection of non-Newtonian nanofluid flow between two vertical flat plates and solved the governing equations numerically by the fourth-order Runge-Kutta method and analytically by the Adomian generalized method. Biswal *et al*. [41] reported the solutions for convective flow of non-Newtonian nanofluid between two vertical parallel plates. They applied two different techniques namely the Galerkin’s method and the homotopy perturbation method to obtain the solutions. The Lie symmetry approach not only unified the existing approaches but also provided a significant extension of these techniques, which ultimately led to the development of the continuous transformation group theory known as the theory of Lie groups. The applications of Lie group approach were thus identified in classical mechanics, quantum mechanics, general relativity, etc. The symmetry analysis was found to provide an algorithmic way of obtaining the similar and self-similar solutions of nonlinear partial differential equations. The unsteady two dimensional flow of magneto-Jaffery fluid in a porous semi infinite channel is studied by Mekheimer *et al*. [42]. The authors applied the Lie group technique for the reductions of partial differential equation and then used the perturbation method. Nchabeleng and Fareo [43], investigated the two dimensional fluid-driven permeable fracture. The Lie symmetry approach is applied in this study and found the group invariant and numerical solutions. The effects of oscillation and radiation for MHD Casson fluid in an asymmetric wavy channel is studied by Tufail *et al*. [44]. The governing equations are solved using the group theoretical method and the effects of different physical parameters of flow and heat transfer are discussed. The application of symmetry approach for solving the non-Newtonian fluid flow problems are not new but it is certainly young enough with scope to expand. Some recent studies in this context are available in [45–51].

In this manuscript, the authors formulate the exact closed-form solutions for a mathematical model of flow and radiative heat transfer of a power-law nanofluid over a moving surface. To the best of the authors’ knowledge, the exact solutions are not reported in the literature for this problem. The flow is generated by the arbitrary motion of the boundary and the Navier’s slip conditions are assumed at the boundary. The effects of linear thermal radiation and transverse applied magnetic field are also included in the model. Tiwari and Das [52] model is adopted for the thermophysical properties of nanofluid. Lie symmetry approach is utilized to find the group invariant solutions. Numerical solutions are also computed and depicted for the *Cu*-water nanofluid to observe the effect of governing thermophysical parameters on the flow velocity and temperature distribution. The commercial application of the present model makes it more considerable for thermal engineering. These applications are linked in the field of nuclear reactors, automotive industry, electronics and medical industry and manufacturing process.

## Problem statement

**Fig 1:**
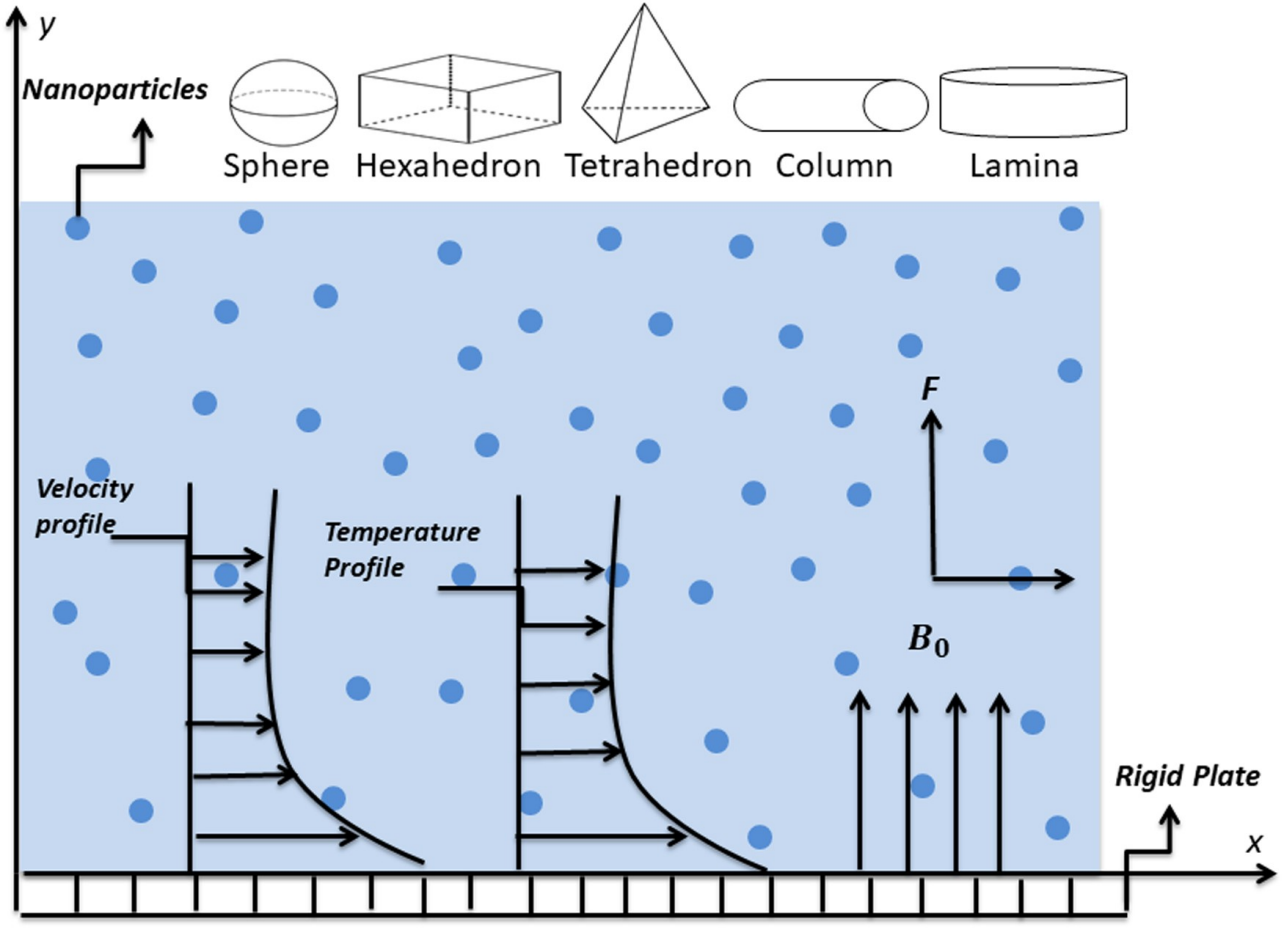
Flow geometry.

Fig 1 represents the unidirectional time-dependent Stokes’ flow and radiative heat transfer of electrically conducting power-law nanofluid. The fluid occupies the space over the infinite rigid surface at *x* = 0. The flow is generated by the arbitrary motion of the rigid surface. The interface of nanofluid and the surface admits the Navier’s slip conditions.

The governing equations for the conservation of mass, momentum and energy under usual boundary layer approximations as given by White [53]:

$$\begin{array}{c} \nabla \cdot F=0, \end{array}$$ (1)

$$\begin{array}{c} \rho_{nf}\frac{DF}{Dt}=-\nabla p+\mu_{nf}\nabla \tau -J\times B, \end{array}$$ (2)

$$\begin{array}{c} {\left(\rho C_{p}\right)}_{nf}\nabla T=\nabla \left(\kappa_{nf}\nabla T\right)-\nabla \cdot q_{r}. \end{array}$$ (3)

In the above equations, **F** is the velocity field, *t* is time, *p* is pressure, **J** is current density, **B** is the magnetic field, *T* is temperature, *q*_*r*_ is radiative heat flux, *ρ*_*nf*_ is density, *μ*_*nf*_ is viscosity, (*C*_*p*_)_*nf*_ is specific heat capacity and *κ*_*nf*_ is the thermal conductivity of nanofluid. The velocity field is assumed as

$$\begin{array}{c} F=(F(y,t),0,0). \end{array}$$ (4)

The continuity Eq (2) together with Eq (4) is identically satisfied. The uniform applied magnetic field have the strength *B*_0_ and is applied in the direction perpendicular to the flow. The induced magnetic field is considered negligible in comparison to the applied magnetic field [54]. Following Bird *et. al*. [55], the shear stress in *x*-direction for the power-law nanofluid is written as:

$$\begin{array}{c} \tau_{xy}=K|F_{y}{|}^{n-1}F_{y}, \end{array}$$ (5)

where *K* is the coefficient of consistency and *n* > 0 is a power-law index. For optically thick nanofluid, the radiative heat can only travel a short distance, therefore the Rosseland approximations are applicable [56, 57]. Under these assumptions, Eqs (2) and (3) take the form

$$\begin{array}{c} \rho_{nf}F_{t}=\mu_{nf}\partial_{y}\tau_{xy}-\sigma_{nf}B_{0}^{2}F, \end{array}$$ (6)

$$\begin{array}{c} {\left(\rho C_{p}\right)}_{nf}T_{t}=\kappa_{nf}T_{yy}+\frac{16\sigma^{*}T_{\infty }^{3}}{3\kappa^{3}}T_{yy}, \end{array}$$ (7)

here *σ*_*nf*_ is electrical conductivity, *T*_∞_ is ambient temperature, *κ* is mean absorption coefficient and σ* is the Stefan Boltzmann constant.

The appropriate boundary conditions for the present model are:

$$\begin{array}{c} F\left(0,t\right)=U_{0}V\left(t\right),\;T\left(0,t\right)=v\left(t\right)+T_{\infty }\;t>0, \end{array}$$ (8)

$$\begin{array}{c} F\left(y,t\right)\to 0,\;T\left(y,t\right)\to T_{\infty }\;\text{as}\;y\to \infty ,\;t>0, \end{array}$$ (9)

$$\begin{array}{c} F\left(y,0\right)=H\left(y\right),\;T\left(y,0\right)=h\left(y\right)+T_{\infty },\;y>0, \end{array}$$ (10)

where *U*_0_ is the reference velocity. The relationship between thermophysical properties of base fluid and nanoparticles are given in Table 1 (for further details see [58–61]).

**Table 1:** Correlations between physical properties of base fluid and nanoparticles.

*Properties*	*Nanofluid*
Density (*ρ*)	*ρ*_*nf*_ = *ρ*_*f*_(1 − *φ*) + *φρ*_*s*_
Viscosity (*μ*)	$\mu_{nf}=\frac{\mu_{f}}{{\left(1-\varphi \right)}^{2.5}}$
Electrical Conductivity (*σ*)	$\sigma_{nf}=\sigma_{f}[1+\frac{3(\frac{\sigma_{s}}{\sigma_{f}}-1)\varphi }{\left(\frac{\sigma_{s}}{\sigma_{f}}+2\right)-\left(\frac{\sigma_{s}}{\sigma_{f}}-1\right)\varphi }]$
Heat Capacity (*ρC*_*p*_)	${\left(\rho C_{p}\right)}_{nf}={\left(\rho C_{p}\right)}_{f}[\left(1-\varphi \right)+\varphi \frac{{\left(\rho C_{p}\right)}_{s}}{{\left(\rho C_{p}\right)}_{f}}]$
Thermal Conductivity (*κ*)	$\kappa_{nf}=\kappa_{f}[\frac{\kappa s+\left(m-1\right)\kappa_{f}-\left(m-1\right)\varphi \left(\kappa_{f}-\kappa_{s}\right)}{\kappa_{s}+\left(m-1\right)\kappa_{f}+\varphi \left(\kappa_{f}-\kappa_{s}\right)}]$

In Table 1, *φ* is the volume concentration of nanoparticles. The parameters *ρ*_*f*_, *μ*_*f*_, *σ*_*f*_, (*ρC*_*p*_)_*f*_ and *κ*_*f*_ are density, viscosity, electrical conductivity, specific heat capacity and thermal conductivity of base fluid respectively. The parameters *ρ*_*s*_, *μ*_*s*_, *σ*_*s*_, (*ρC*_*p*_)_*s*_ and *κ*_*s*_ are density, viscosity, electrical conductivity, specific heat capacity and thermal conductivity of nanoparticles. The shape factor *m* is explained in Table 2 (see details in Jamshed and Aziz [62]).

**Table 2:** Values of empirical shape factor for different particle shapes.

*Nanoparticle* *shapes*	*Sphere*	*Hexahedron*	*Tetrahedron*	*Column*	*Lamina*
*m*	3	3.7221	4.0613	6.3698	16.1576

To establish dimensionless form of the Eqs (6)–(10), the following non-dimensional parameters are introduced

$$\begin{array}{c} \bar{F}=\frac{F}{U_{0}},\;\bar{t}=\frac{U_{0}}{L}t,\;\bar{y}=(\frac{\rho_{f}U_{0}^{2-n}L^{n}}{K}{)}^{\frac{1}{n-1}}\frac{y}{L},\\ {\bar{\tau }}_{\bar{x}\bar{y}}=|F_{y}{|}^{n-1}F_{y}=(\frac{KU_{0}^{3-n}\rho_{f}}{L^{n}}{)}^{\frac{-1}{n+1}}\tau_{xy},\;\sigma_{f}B_{0}^{2}={\bar{M}}^{2}\frac{\rho_{f}}{L}U_{0},\;\bar{T}=\frac{T-T_{\infty }}{T_{w}-T_{\infty }}. \end{array}$$ (11)

Using the relations given in Eq (11) and neglecting bars, Eqs (6)–(10) take the form

$$\begin{array}{c} F_{t}=\mu_{*}\partial_{y}(|F_{y}{|}^{n-1}F_{y})-M_{*}F, \end{array}$$ (12)

$$\begin{array}{c} T_{t}=\frac{1}{{\left(\rho C_{p}\right)}_{*}P_{r}}([\frac{\kappa s+\left(m-1\right)\kappa_{f}-\left(m-1\right)\varphi \left(\kappa_{f}-\kappa_{s}\right)}{\kappa_{s}+\left(m-1\right)\kappa_{f}+\varphi \left(\kappa_{f}-\kappa_{s}\right)}]+N_{r})T_{yy}, \end{array}$$ (13)

$$\begin{array}{c} F(0,t)=V(t),\;T(0,t)=v(t)\;t>0, \end{array}$$ (14)

$$\begin{array}{c} F(y,t)\to 0,\;T(y,t)\to 0\;\text{as}\;y\to \infty ,\;t>0, \end{array}$$ (15)

$$\begin{array}{c} F(y,0)=H(y),\;T(y,0)=h(y),\;y>0. \end{array}$$ (16)

In the above system, $\mu_{*}=\frac{1}{\varphi_{*}{\left(1-\varphi \right)}^{2.5}}$ is flow parameter, $M_{*}=\frac{\sigma_{*}M^{2}}{\varphi_{*}}$ is magnetic parameter, $P_{r}=\frac{\kappa_{f}}{\rho_{f}^{\frac{n-1}{n+2}}U_{0}^{\frac{3(n-1)}{n+1}}L^{\frac{1-n}{1+n}}K^{\frac{2}{n+1}}{\left(C_{p}\right)}_{f}}$ is local Prandtl number, $N_{r}=\frac{16T_{\infty }^{3}\sigma_{*}}{3\kappa^{*}\kappa_{f}}$ is thermal radiation parameter, with $\varphi_{*}=(1-\varphi +\varphi \frac{\rho_{s}}{\rho_{f}})$ and ${\left(\rho C_{p}\right)}_{*}=(\left(1-\varphi \right)+\varphi \frac{{\left(\rho C_{p}\right)}_{s}}{{\left(\rho C_{p}\right)}_{f}})$. The boundary conditions in Eq (14) indicate the Navier slip condition because the fluid particles attached with the boundary are moving with a specific velocity.

## Newtonian nanofluid flow model: A case study

We first discuss the special case of the model by considering the Newtonian nanofluid problem. In this case Eq (12) takes the form

$$\begin{array}{c} F_{t}=\mu_{*}F_{yy}-M_{*}F. \end{array}$$ (17)

The following transformation

$$\begin{array}{c} \tilde{F}\left(y,t\right)=F\left(y,t\right)\text{exp}(-M_{*}t), \end{array}$$ (18)

reduces Eq (17) to

$$\begin{array}{c} {\tilde{F}}_{t}=\mu_{*}{\tilde{F}}_{yy}. \end{array}$$ (19)

The use of transformation (18) in boundary and initial conditions (14)–(16) gives

$$\begin{array}{c} \tilde{F}\left(0,t\right)=V\left(t\right),\;t>0,\;\tilde{F}\left(\infty ,t\right)=0,\;t>0,\;\tilde{F}\left(y,0\right)=H\left(y\right),\;y>0, \end{array}$$ (20)

where *V*(*t*) = *v*(*t*) exp(−M_*_ *t*). Thus, the problem of Newtonian nanofluid flow reduces to the Cauchy problem of the heat equation. To form the closed-form analytical solution of the Cauchy problem (19) and (20), we make use of the results for Cauchy problem of the classical heat equation. The standard heat equation of the Cauchy problem is given by

$$\begin{array}{c} {\tilde{F}}_{t}={\tilde{F}}_{yy},\;\text{with}\;\tilde{F}\left(y,0\right)=\Phi \left(y\right), \end{array}$$ (21)

for some “well-behaved” function Φ, the fundamental solution of the problem (21) is (Hadamard, [63]) and is given by

$$\begin{array}{c} \tilde{F}\left(y,t\right)=\frac{1}{2\sqrt{t\pi }}\int_{-\infty }^{+\infty }\Phi \left(\zeta \right)\text{exp}[-\frac{{\left(y-\zeta \right)}^{2}}{4t}]d\zeta . \end{array}$$ (22)

The solution (22) can be transformed into the solution of (17) after using transformation (18). The substitution of $\tilde{F}\left(y,t\right)$ from Eq (22) into Eq (18), gives

$$\begin{array}{c} F\left(y,t\right)=\frac{\mu_{*}}{2\sqrt{t\pi }}\text{exp}(M_{*}t)\int_{-\infty }^{+\infty }\Phi \left(\zeta \right)\text{exp}[-\frac{{\left(y-\zeta \right)}^{2}}{4t}]d\zeta , \end{array}$$ (23)

where Φ(*ζ*) = *H*(*ζ*). Here *V*(*t*) is given by

$$\begin{array}{c} \bar{V}\left(t\right)=\frac{\mu_{*}}{2\sqrt{t\pi }}\text{exp}(M_{*}t)\int_{-\infty }^{+\infty }\Phi \left(\zeta \right)\text{exp}[-\frac{\zeta^{2}}{4t}]d\zeta . \end{array}$$ (24)

The integral in Eq (24) can be solved in terms of the Kummer confluent hypergeometric functions.

## Lie symmetry analysis

A symmetry of a differential equation (DE) is a transformation of dependent and independent variables. This is an invertible transformation that maps an equation to itself. The procedure to find a local one-parameter group of transformation is available in literature (see details in [64–67]). In case of PDEs, the symmetries reduces the number of independent variables and transform PDE into an ODE.

Following procedure is adopted to find the local one-parameter group of transformation. An infinitesimal transformation can be written as

$$\begin{array}{c} \bar{t}\approx t+\epsilon \tau_{1}\left(t,y,F\right)+\cdots ,\\ \bar{y}\approx y+\epsilon \tau_{2}\left(t,y,F\right)+\cdots ,\\ \bar{F}\approx F+\epsilon \tau_{3}\left(t,y,F\right)+\cdots , \end{array}$$ (25)

here *ϵ* is a small group parameter and it is a Lie point symmetry of Eq (12) if and only if

$$\begin{array}{c} \Omega^{\left[2\right]}{\left|F_{t}-\mu_{*}\partial_{y}(|F_{y}{|}^{n-1}F_{y})+M_{*}F\right|}_{Eq.(12)}=0, \end{array}$$ (26)

where

$$\begin{array}{c} \Omega^{\left[2\right]}=\tau_{1}\partial_{t}+\tau_{2}\partial_{y}+\tau_{3}\partial_{F}+\tau_{3}^{t}\partial_{F_{t}}+\tau_{3}^{y}\partial_{F_{y}}+\tau_{3}^{yy}\partial_{F_{yy}}, \end{array}$$ (27)

with

$$\begin{array}{cc} {\tau_{3}}^{t} & =D_{t}\tau_{3}-F_{t}D_{t}\tau_{1}-F_{y}D_{t}\tau_{2},\\ {\tau_{3}}^{y} & =D_{y}\tau_{3}-F_{t}D_{y}\tau_{1}-F_{y}D_{y}\tau_{2},\\ {\tau_{3}}^{yy} & =D_{y}\tau_{3}^{y}-F_{ty}D_{y}\tau_{1}-F_{yy}D_{y}\tau_{2}. \end{array}$$ (28)

The total derivative operators are given by

$$\begin{array}{c} D_{t}=\partial_{t}+F_{t}\partial_{F}+F_{tt}\partial_{F_{t}}+F_{ty}\partial_{F_{y}}+\ldots , \end{array}$$ (29)

$$\begin{array}{c} D_{y}=\partial_{y}+F_{t}\partial_{F}+F_{yy}\partial_{F_{y}}+F_{ty}\partial_{F_{t}}+\ldots . \end{array}$$ (30)

In Eq (26), the undetermined functions *τ*_1_, *τ*_2_ and *τ*_3_ are independent of the derivatives of *F*. Thus by separating this equation with respect to derivatives and their powers gives the over-determined system of linear partial differential equations as follows

$$\begin{array}{cc} {\tau_{1}}_{y}={\tau_{1}}_{F}={\tau_{2}}_{F}={\tau_{2}}_{t}={\tau_{3}}_{y}={\tau_{3}}_{FF} & =0,\\ {\tau_{3}}_{t}+M_{*}\left(\tau_{3}+F\left(-{\tau_{3}}_{F}+{\tau_{1}}_{t}\right)\right) & =0,\\ n\mu_{*}\left(\left(-1+n\right){\tau_{3}}_{F}+{\tau_{1}}_{t}-{\tau_{2}}_{y}-n{\tau_{2}}_{y}\right) & =0. \end{array}$$ (31)

The above system can be solved for different values of *n* which gives the different symmetry generator in each case.

**Case I**: *n* = 1, μ_*_ ≠ 0, M_*_ ≠ 0

In this case, the following six dimensional Lie algebra is obtained as

$$\begin{array}{c} \Omega_{1}=\partial_{t},\;\Omega_{2}=\partial_{y},\;\Omega_{3}=2t\partial_{t}+y\partial_{y}-2FtM_{*}\partial_{F},\\ \Omega_{4}=e^{-tM_{*}}y\partial_{F},\;\Omega_{5}=e^{-tM_{*}}\partial_{F},\;\Omega_{6}=F\partial_{F}. \end{array}$$ (32)

**Case II**: *n* ≠ 1, μ_*_ ≠ 0, M_*_ ≠ 0

This case specifies the non-Newtonian fluid and the six dimensional algebra is found as

$$\begin{array}{c} \Omega_{1}=\partial_{t},\;\Omega_{2}=\partial_{y},\;\Omega_{3}=y\partial_{y}+(\frac{n+1}{n-1})F\partial_{F},\;\Omega_{4}=2t\partial_{t}+y\partial_{y}+F\partial_{F},\\ \Omega_{5}=\text{exp}(-M_{*}t)\partial_{F},\;\Omega_{6}=\frac{1}{M_{*}}(-\text{exp}(M_{*}\left(n-1\right)t)\partial_{t}+\text{exp}(M_{*}\left(n-1\right)t)F\partial_{F}). \end{array}$$ (33)

The symmetries for the energy Eq (13) can be found under the same methodology as observed for the flow equation. The symmetry condition for the Eq (13) is

$$\begin{array}{c} \Omega^{\left[2\right]}{\left|T_{t}-\frac{1}{{\left(\rho C_{p}\right)}_{*}P_{r}}([\frac{\kappa s+\left(m-1\right)\kappa_{f}-\left(m-1\right)\varphi \left(\kappa_{f}-\kappa_{s}\right)}{\kappa_{s}+\left(m-1\right)\kappa_{f}+\varphi \left(\kappa_{f}-\kappa_{s}\right)}]-N_{r})T_{yy}\right|}_{Eq.(13)}=0, \end{array}$$ (34)

where Ω^[2]^ is given in Eq (27) and various expressions therein are the same as given in Eqs (28)–(30) by replacing *F* with *T*. The separation of derivatives and powers of derivative of *T* gives the following over-determined system of linear partial differential equations as

$$\begin{array}{c} {\tau_{1}}_{y}={\tau_{1}}_{TT}={\tau_{2}}_{TT}={\tau_{1}}_{T}=0,\;{\tau_{3}}_{TT}-2{\tau_{2}}_{yT}=0,\\ P_{r}{\left(\rho C_{p}\right)}_{*}{\tau_{3}}_{t}-\left(N_{r}+\kappa_{*}\right){\tau_{3}}_{yy}=0,\;(P_{r}{\left(\rho C_{p}\right)}_{*}{\tau_{2}}_{T}+\left(N_{r}+\kappa_{*}\right){\tau_{1}}_{yT}=0,\\ P_{r}{\left(\rho C_{p}\right)}_{*}{\tau_{2}}_{t}+\left(N_{r}+\kappa_{*}\right)\left(2{\tau_{3}}_{yT}-{\tau_{2}}_{yy}\right)=0,\\ -P_{r}{\left(\rho C_{p}\right)}_{*}\left({\tau_{1}}_{t}-2{\tau_{2}}_{y}\right)+\left(N_{r}+\kappa_{*}\right){\tau_{1}}_{yy}=0, \end{array}$$ (35)

here $\kappa_{*}=[\frac{\kappa s+\left(m-1\right)\kappa_{f}-\left(m-1\right)\varphi \left(\kappa_{f}-\kappa_{s}\right)}{\kappa_{s}+\left(m-1\right)\kappa_{f}+\varphi \left(\kappa_{f}-\kappa_{s}\right)}]$ is chosen for convenience. The above system of governing equations gives the infinite dimensional Lie algebra generated by

$$\begin{array}{c} \Omega_{1}=\partial_{t},\;\Omega_{2}=\partial_{y},\;\Omega_{3}=t\partial_{t}+\frac{y}{2}\partial_{y},\\ \Omega_{4}=t^{2}\partial_{t}+ty\partial_{y}+(-\frac{tT}{2}-\frac{P_{r}{\left(\rho C_{p}\right)}_{*}Ty^{2}}{4(N_{r}+\kappa_{*})})\partial_{T},\\ \Omega_{5}=t\partial_{y}-\frac{P_{r}{\left(\rho C_{p}\right)}_{*}Ty}{2(N_{r}+\kappa_{*})}\partial_{T},\;\Omega_{6}=T\partial_{T},\;\Omega_{7}=\Gamma \left(y,t\right)\partial_{T}, \end{array}$$ (36)

where the function Γ(*y*, *t*) satisfies the equation

$$\begin{array}{c} P_{r}{\left(\rho C_{p}\right)}_{*}\Gamma_{t}-N_{r}\Gamma {\left(y,t\right)}_{yy}-\kappa_{*}\Gamma {\left(y,t\right)}_{yy}=0. \end{array}$$

## Group invariant solutions for flow model

In this section, the exact and numerical solutions to the problem formulated in section 4 are found corresponding to their infinitesimal generators. Separate solutions are presented for the Newtonian and non-Newtonian flow models. Graphs are also produced for the *Cu*-water nanofluid to illustrate the effect of governing physical parameters.

### Solution via subgroup generated by Ω_4_ in case II

The similarity variable corresponding to operator Ω_4_ is assumed as

$$\begin{array}{c} F\left(y,t\right)=\sqrt{t}f_{1}\left(\xi \right),\;\xi =\frac{y}{\sqrt{t}}. \end{array}$$ (37)

Substitution of Eq (37) in Eq (12), gives a differential equation in *f*_1_(*ξ*), that is

$$\begin{array}{c} n\mu_{*}{\left(f_{1}^{\prime }\right)}^{n-1}f_{1}^{\prime \prime }+\frac{\xi }{2}f_{1}^{\prime }-(\frac{1}{2}+M_{*})f_{1}=0. \end{array}$$ (38)

The boundary conditions (14)–(16) transform to

$$\begin{array}{c} f_{1}\left(0\right)=p_{0},\;f_{1}\left(\xi \right)\to 0\;as\;\xi \to \infty , \end{array}$$ (39)

where *p*_0_ is some constant. The group-compatibility approach (see details in Aziz *et al*. [68]), forms the exact solution of Eq (38) for *n* = 1 and is given as

$$\begin{array}{c} f_{1}\left(\xi \right)=p_{0}\text{exp}(-\gamma \xi ), \end{array}$$ (40)

where *γ* is given by

$$\begin{array}{c} \gamma =\frac{\frac{\xi }{2}\pm \sqrt{\frac{\xi^{2}}{4}+4\mu_{*}\left(\frac{1}{2}+M_{*}\right)}}{2\mu_{*}}. \end{array}$$ (41)

The backward substitution form the solution of PDE (12) as

$$\begin{array}{c} F\left(y,t\right)=\sqrt{t}\;\text{exp}[-(\frac{\frac{y}{2\sqrt{t}}\pm \sqrt{\frac{y^{2}}{4t}+4\mu_{*}\left(\frac{1}{2}+M_{*}\right)}}{2\mu_{*}})\frac{y}{\sqrt{t}}]. \end{array}$$ (42)

The above solution satisfies the boundary conditions in Eqs (14)–(16) with $V\left(t\right)=\sqrt{t}$ and *H*(*y*) = 0.

To find the numerical solution of Eq (38) along with the initial and boundary conditions (39) for *n* ≠ 1, MATLAB bvp4c numerical solver is used. In order to use the bvp4c solver one has to convert Eq (37) into a system of first order ordinary differential equations, that is

$$\begin{array}{c} f_{1}^{\prime }=q,\;q^{\prime }=\frac{\left(\frac{1}{2}+M_{*}\right)f_{1}-\frac{\xi }{2}q}{n\mu_{*}q^{n-1}}, \end{array}$$ (43)

$$\begin{array}{c} f_{1}\left(\xi \right)=p_{0},\;at\;\xi =0,\;f_{1}\left(\xi \right)\to 0\;as\;\xi \to \infty . \end{array}$$ (44)

The system is solved using the appropriate initial values of *q*(*ξ*) at *ξ* = 0.

**Fig 2:**
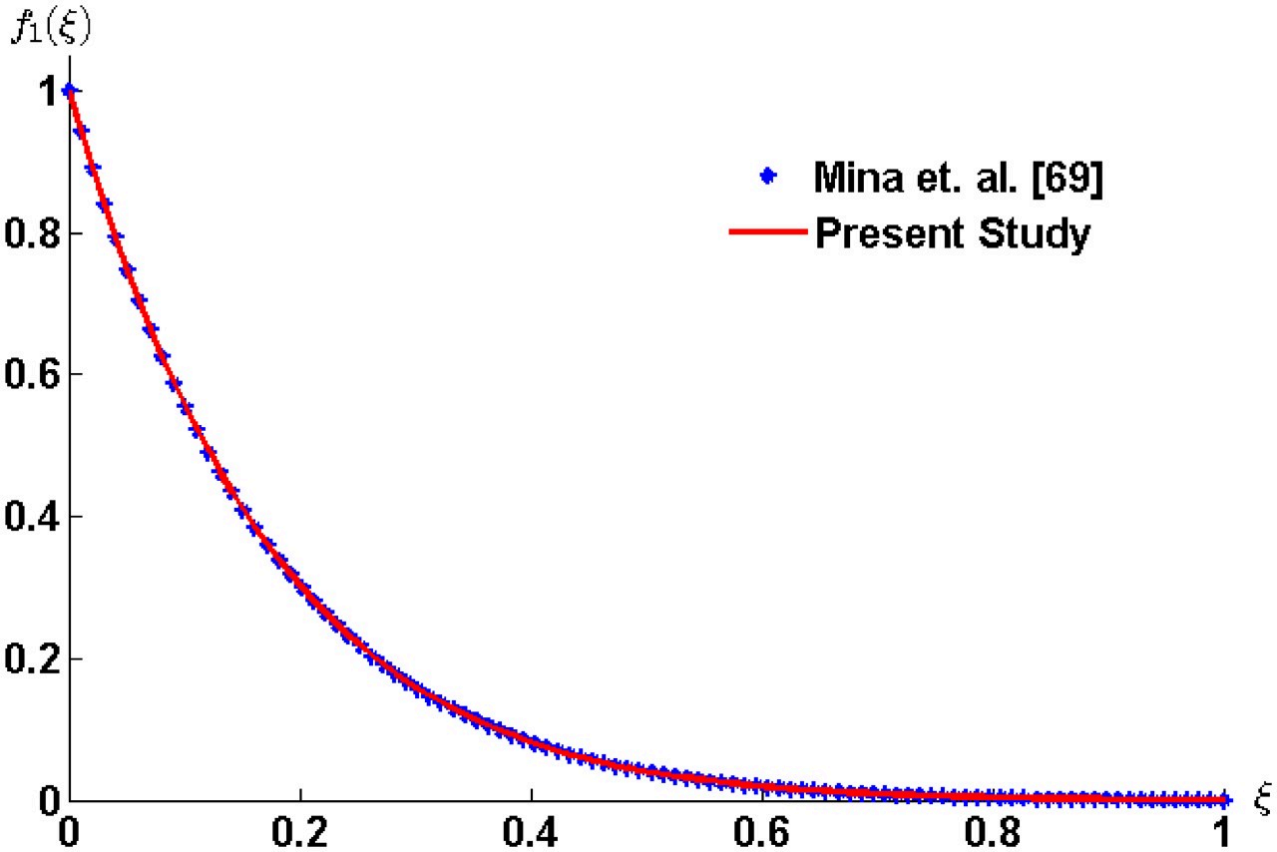
Comparison with Mina *et al*. [69] in the absence of nanoparticles and for *n* = 1.

To validate the obtained results comparison is made from the results available in literature. The ordinary differential equation (5.4) of [69] and Eq (38) in the absence of nanoparticles of the present study are plotted numerically using the bvp4c solver. The graphs shown in Fig 2 shows the accuracy of the results.

#### Graphical results and discussion

Here, the exact and numerical solutions are depicted to examine the effect of different physical parameters on the velocity of power-law nanofluid. The graphs are produced for the water based *Cu*-water nanofluid. The thermophysical properties of cu-water nanofluid can be found in Aziz and Jamshed [24].

**Fig 3:**
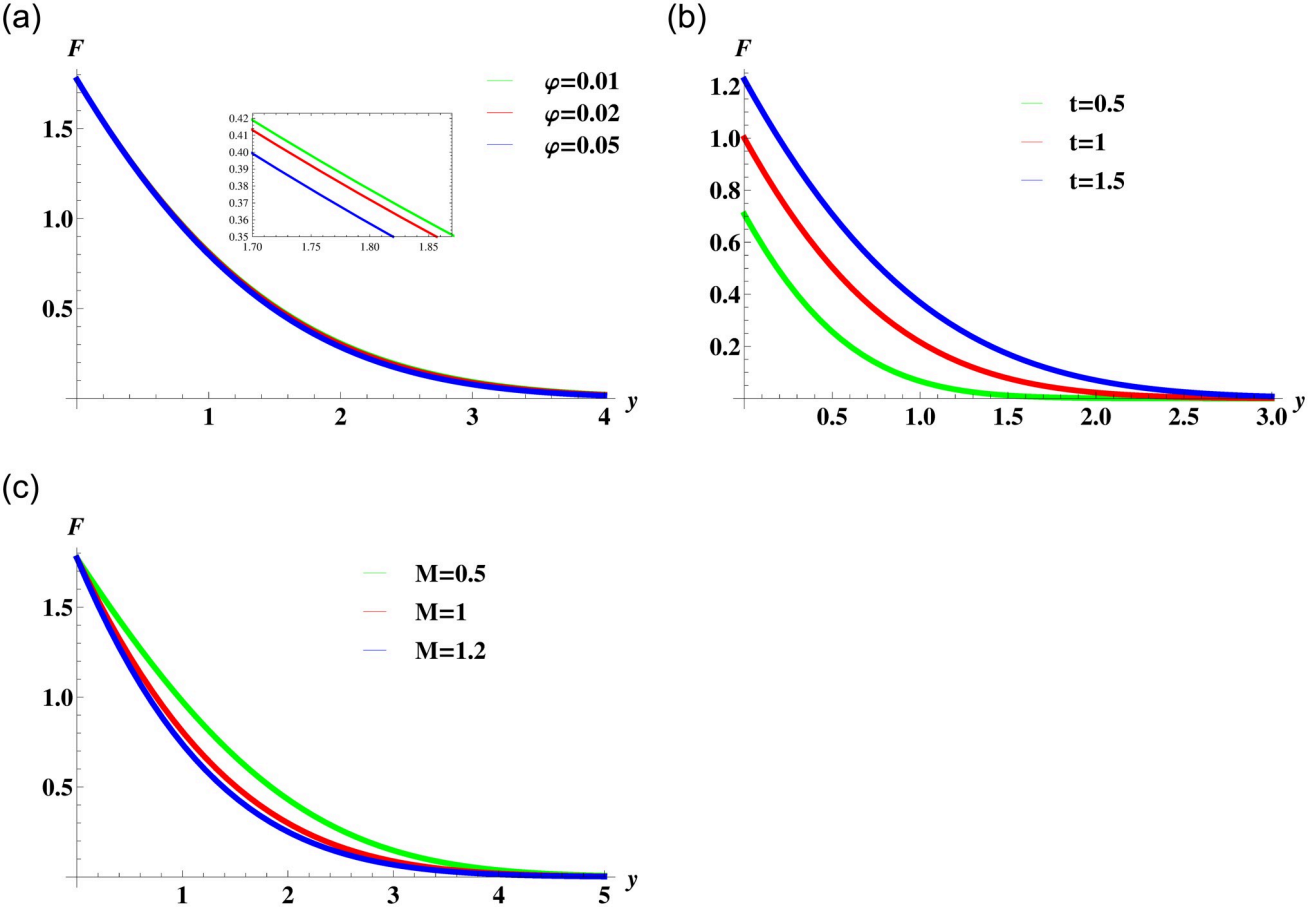
Velocity profile (42) for various values of *φ*, *t* and *M* when *ρ*_*s*_ = 8933, *ρ*_*f*_ = 997.1 *σ*_*s*_ = 5.5 × 10^−6^ and *σ*_*f*_ = 5.96 × 10^7^ are fixed.

Fig 3(*a*)–3(*c*) display the behaviour of Newtonian nanofluid velocity affected by the variation in nanoparticles concentration in the base fluid, time and the magnetic field strength. The Fig 3(a) illustrates that the nanofluid motion retards with an increase in the concentration of nanoparticles. The solid nanoparticles are dense and an increase in their concentration causes an increase in the overall viscosity of the fluid. This in turn reduces the fluid motion within the boundary layer and causes a reduction of the momentum boundary layer. It is also clear from the figure that the nanofluid attains maximum velocity at the boundary that gradually decreases to the free stream velocity. This satisfies the assumed boundary conditions and also verifies the validity of the solution. Fig 3(*b*) shows the velocity of nanofluid increases with time and causes thickening of the momentum boundary layer. The transverse magnetic field produces a resistive Lorentz force in the nanofluid. The increase in the strength of fluid resistance is evident by the decreasing trend in the nanofluid velocity due to the augmentation of the magnetic field parameter in Fig 3(*c*).

**Fig 4:**
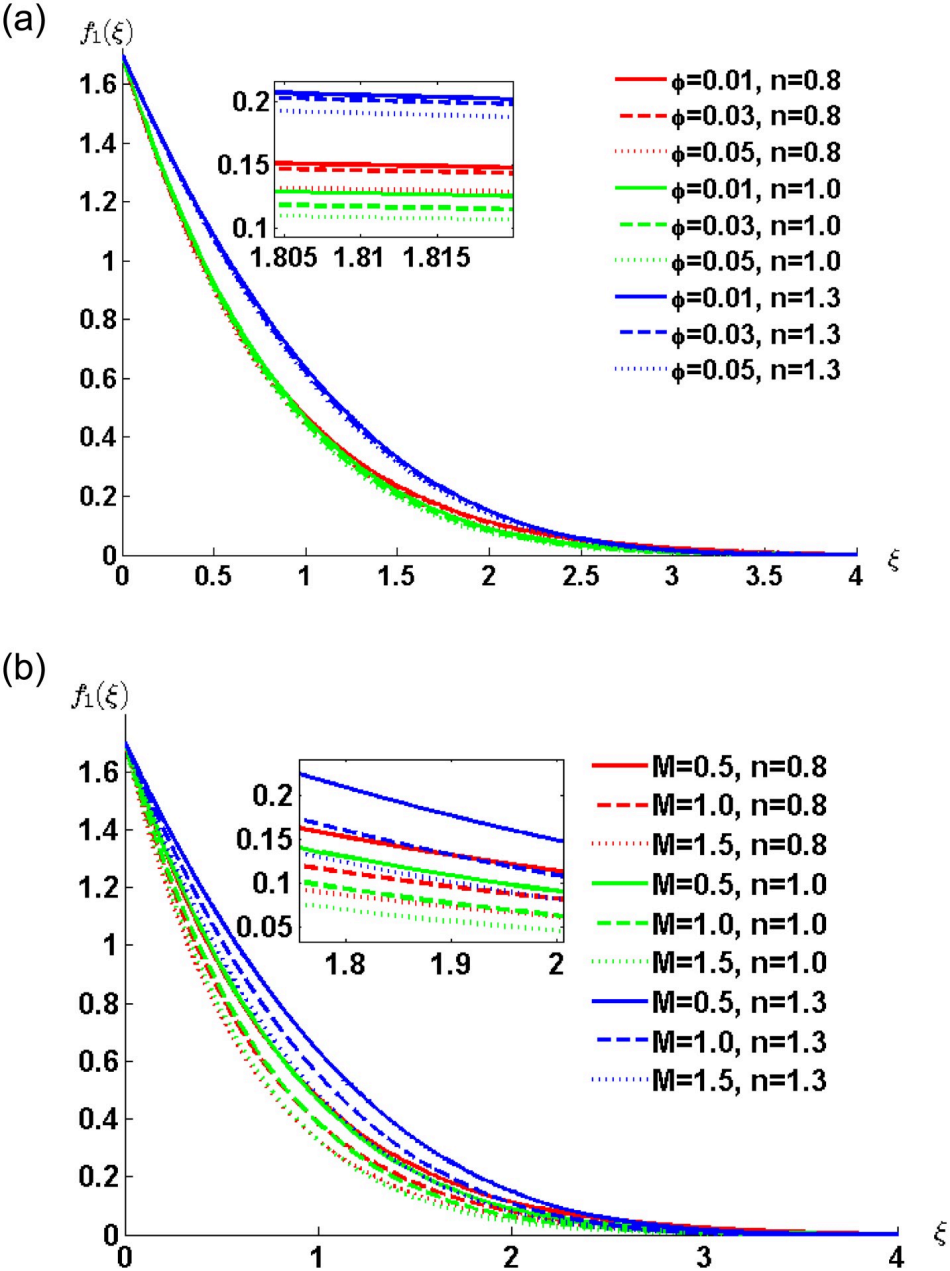
Velocity profile for various values of *φ* and *M* when *ρ*_*s*_ = 8933, *ρ*_*f*_ = 997.1 *σ*_*s*_ = 5.5 × 10^−6^ and *σ*_*f*_ = 5.96 × 10^7^ are fixed.

Fig 4(*a*) and 4(*b*) illustrates the combined effect of power-law index *n*, nanoparticles volume concentration and magnetic field on the nanofluid velocity. The curves in Fig 4(*a*) show decreasing trend in fluid velocity for the Newtonian (*n* = 1), shear thinning (*n* < 1) and shear thickening (*n* > 1) nanofluids. The presence of nanoparticles leads to an increase in nanofluid viscosity which in turn creates resistance to the fluid motion. Moreover, for the particular value of parameter *φ* initially the velocity of shear thickening fluid is highest followed by the Newtonian fluid and then the shear thinning fluid. This is due to the initial movement of the boundary. Fig 4(*b*) elucidates the effect of magnetic parameter on the fluid motion for different values of the power-law index. The general behaviour of nanofluid motion is consistent with the fact that the applied magnetic field resists the fluid motion.

### Solution via subgroup generated by Ω_1_ + *α*Ω_4_ in case II

In this subsection, Eq (12) is reduced to an ordinary differential equation under the operator

$$\begin{array}{c} \Omega =\Omega_{1}+\alpha \Omega_{4}=\left(1+2t\alpha \right)\frac{\partial }{\partial t}+\alpha (y\frac{\partial }{\partial y}+F\frac{\partial }{\partial F}), \end{array}$$ (45)

where *α* ∈ ***R***. The invariants corresponding to Eq (45) are given by

$$\begin{array}{c} F\left(y,t\right)=\sqrt{1+2\alpha t}f_{2}\left(\xi \right),\;\xi =\frac{y}{\sqrt{1+2t\alpha }}. \end{array}$$ (46)

Substituting Eq (46) in Eq (12) results in

$$\begin{array}{c} n\mu_{*}{\left(f_{2}^{\prime }\right)}^{n-1}f_{2}^{\prime \prime }+\alpha \xi f_{2}^{\prime }-\left(\alpha +1\right)M_{*}f_{2}=0, \end{array}$$ (47)

with conditions that follow from Eqs (14)–(16) are:

$$\begin{array}{c} f_{2}\left(0\right)=r_{0}\text{,}\;\;f_{2}\left(\xi \right)\to 0\;\;\;\text{as}\;\;\;\xi \to \infty . \end{array}$$ (48)

By making use of the general compatibility analysis [68], the solution of the reduced ODE (47) subject to the boundary conditions (48) for *n* = 1, is given by

$$\begin{array}{c} f_{2}\left(\xi \right)=r_{0}\;\text{exp}[-(\frac{\alpha \xi \pm \sqrt{\alpha^{2}\xi^{2}+4\mu_{*}M_{*}\left(\alpha +1\right)}}{2\mu_{*}})\xi ]. \end{array}$$ (49)

This results in the solution of Eq (46) as

$$\begin{array}{c} F\left(y,t\right)=\sqrt{1+2t\alpha }\;\text{exp}[-(\frac{\frac{\alpha y}{\sqrt{1+2t\alpha }}\pm \sqrt{4\mu_{*}M_{*}\left(\alpha +1\right)+\frac{\alpha^{2}y^{2}}{1+2t\alpha }}}{2\mu_{*}})\frac{y}{\sqrt{1+2t\alpha }}], \end{array}$$ (50)

with condition that $4\mu_{*}M_{*}\left(\alpha +1\right)+\frac{\alpha^{2}y^{2}}{1+2t\alpha }\geq 0$. We note that the solution (50) satisfies the initial and boundary conditions (14)–(16) with

$$\begin{array}{c} F\left(0,t\right)=V\left(t\right)=\sqrt{1+2t\alpha }\;\;\;\text{and}\;\;\;F\left(y,0\right)=H\left(y\right)=0. \end{array}$$ (51)

To approximate the numerical solution of Eq (47) for *n* ≠ 1 using MATLAB bvp4c, Eq (47) is converted into the system

$$\begin{array}{c} f_{2}^{\prime }=q,\;q^{\prime }=\frac{\left(\alpha +1\right)M_{*}f_{2}-\alpha \xi q}{n\mu_{*}q^{n-1}}, \end{array}$$ (52)

$$\begin{array}{c} f_{2}\left(\xi \right)=r_{0},\;at\;\xi =0,\;f_{2}\left(\xi \right)\to 0\;as\;\xi \to \infty , \end{array}$$ (53)

and initial guesses are taken by using collocation method for *q*(*ξ*) at *ξ* = 0.

**Fig 5:**
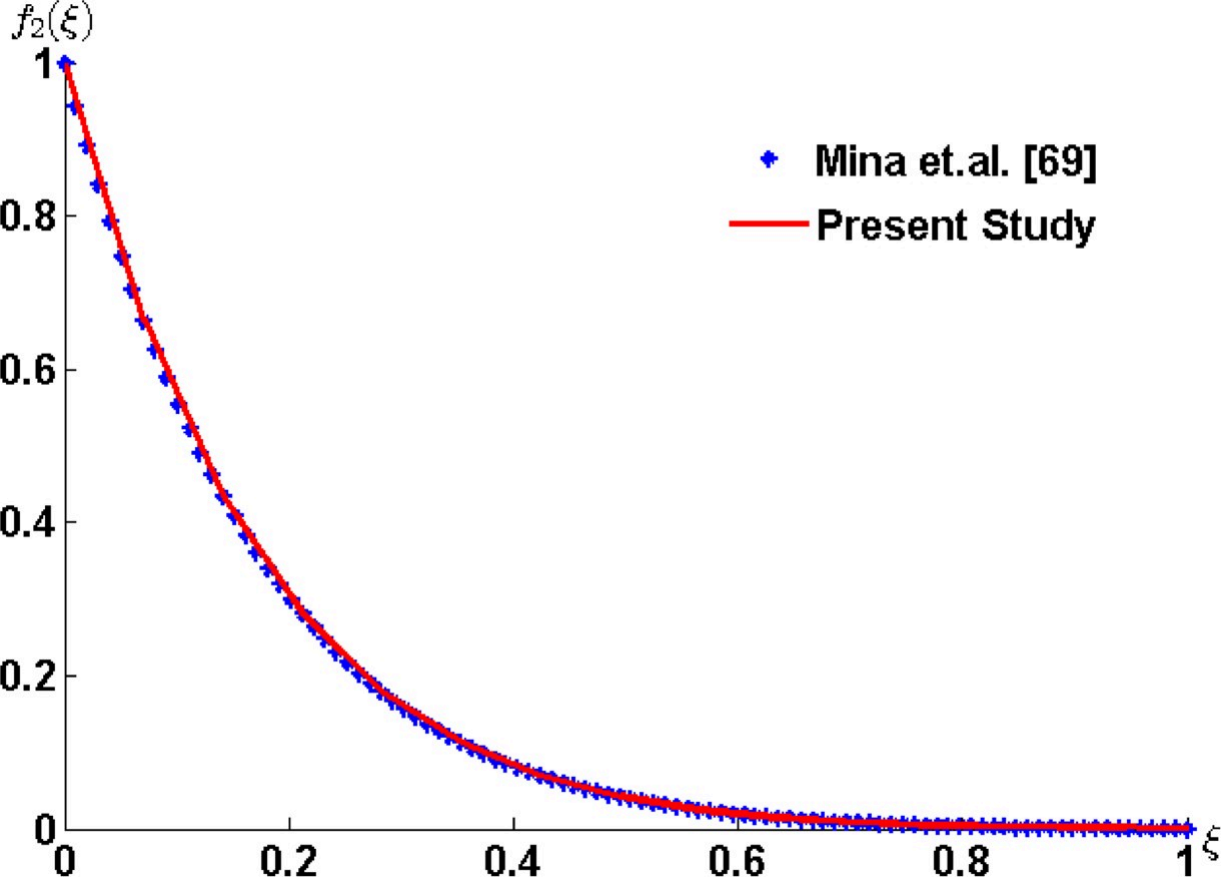
Comparison with Mina *et al*. [69] in the absence of nanoparticles and for *n* = 1, *α* = 0.5, M_*_ = 2 and μ_*_ = 0.1.

The comparison of the velocity profile is made to show the accuracy of the results that is available in the literature. The ordinary differential equation (5.4) of [69] and Eq (47) in the absence of nanoparticles of the present study are plotted numerically using the bvp4c solver. The results given in Fig 5 shows the exact match.

#### Graphical results and discussion

**Fig 6:**
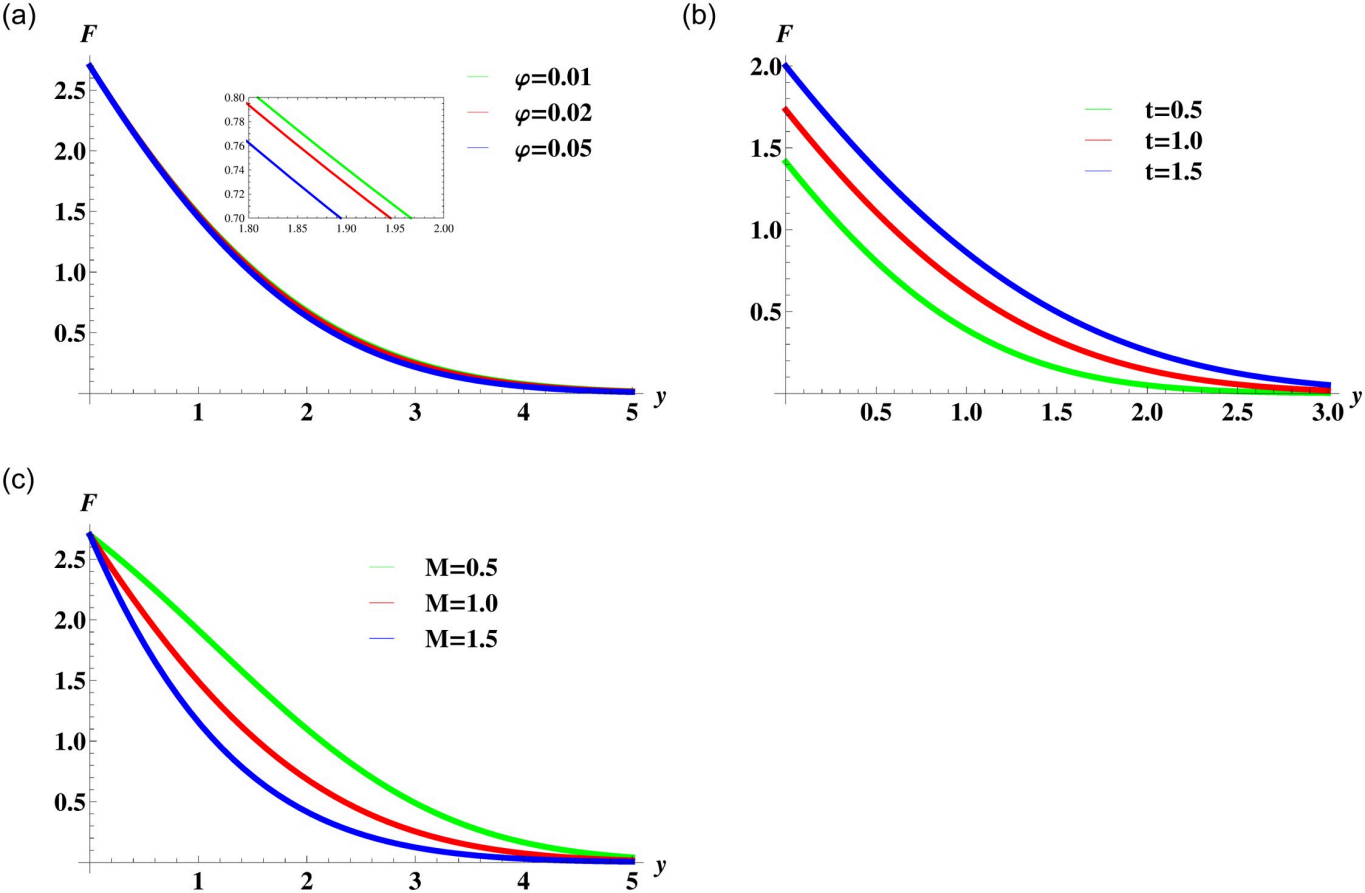
Velocity profile (50) for various values of *φ*, *t* and *M* when *ρ*_*s*_ = 8933, *ρ*_*f*_ = 997.1 *σ*_*s*_ = 5.5 × 10^−6^ and *σ*_*f*_ = 5.96 × 10^7^ are fixed.

In Fig 6(*a*)–6(*c*), the solution for nanofluid velocity given in Eq (50) is plotted to study the effect of variation in the nanoparticles volume concentration, time and the strength of the magnetic field. The general behaviour is consistent with the fact that the increase in the concentration of nanoparticles and the strength of resistive Lorentz force reduces the fluid motion in the boundary layer region. Fig 6(*b*) shows that with time the fluid velocity increases and slowly it comes to the surface.

**Fig 7:**
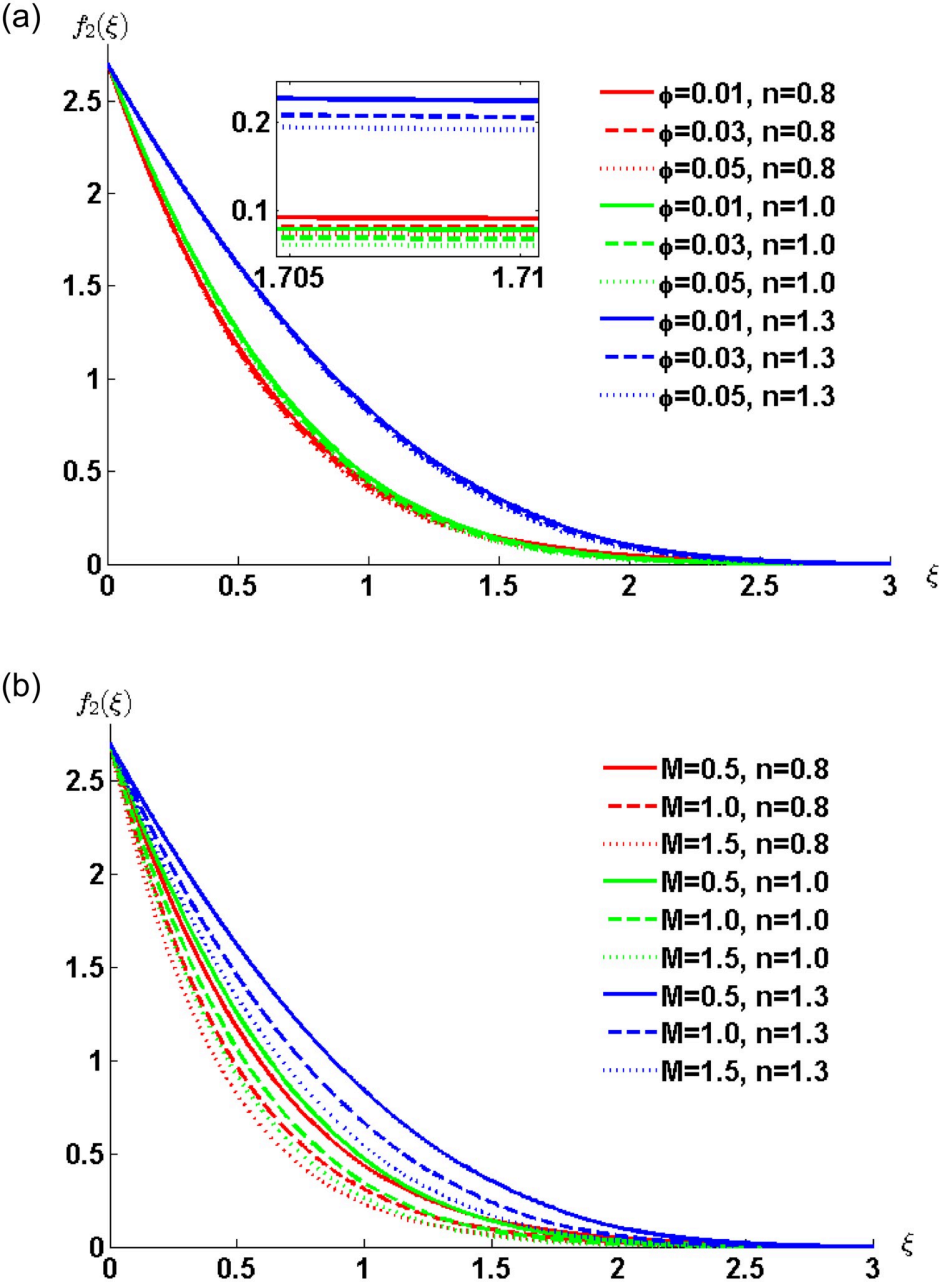
Velocity profile for various values of *φ*, and *M* when *ρ*_*s*_ = 8933, *ρ*_*f*_ = 997.1 *σ*_*s*_ = 5.5 × 10^−6^ and *σ*_*f*_ = 5.96 × 10^7^ are fixed.

Fig 7(*a*) and 7(*b*) depict the effect of nanoparticles volume concentration and the magnetic parameters respectively. Numerical computations are performed for different values of power-law index *n*. Fig 7(*a*) shows that the fluid velocity decreases with increasing concentration of nanoparticles, which causes the thinning of the momentum boundary layer. The thinnest momentum boundary layer is observed for the dilatant nanofluid. The general behavior of resistive Lorentz force can be observed from Fig 7(*b*). At any particular time the lowest velocity is for the shear thinning fluid followed by the Newtonian and shear thickening fluid. The fluid comes near to the surface rapidly which causes the momentum boundary layer to become thick.

### Solution via subgroup generated by Ω_1_ − *c*Ω_2_

The linear combination of time and space translation operators in Eqs (32) and (33) can be written as

$$\begin{array}{c} \Omega =\partial_{t}-c\partial_{y},\;c>0, \end{array}$$ (54)

where *c* is the wave speed. The Eq (54) gives travelling wave solution for the fluid flow. The invariants are found as

$$\begin{array}{c} F\left(y,t\right)=f_{3}\left(\xi \right),\;\xi =y+ct,\;c>0. \end{array}$$ (55)

The substitution of Eq (55) into Eq (12) reduces it to an ODE in *f*_3_(*ξ*), that is

$$\begin{array}{c} cf_{3}^{\prime }-n\mu_{*}(f_{3}^{\prime }{)}^{n-1}f_{3}^{\prime \prime }-M_{*}f_{3}=0. \end{array}$$ (56)

The boundary conditions are transformed to

$$\begin{array}{c} f_{3}\left(0\right)=1,\;f_{3}\left(\xi \right)\to 0\;\text{as}\;\xi \to \infty . \end{array}$$ (57)

Following the same procedure used in the previous sections, the solution of boundary value problem (56)-(57) for *n* = 1 is

$$\begin{array}{c} f_{3}\left(\xi \right)=\text{exp}(-\beta \xi ), \end{array}$$ (58)

with *β* is given by

$$\begin{array}{c} \beta =\frac{\left[-c\pm \sqrt{c^{2}+4M_{*}\mu_{*}}\right]}{2\mu_{*}}. \end{array}$$ (59)

For real solution, we have the condition that *c*^2^ + 4M_*_ μ_*_ ≥ 0. Therefore *β* is equal to

$$\begin{array}{c} \beta_{1}=\frac{\left[-c+\sqrt{c^{2}+4M_{*}\mu_{*}}\right]}{2\mu_{*}},\;\text{or}\;\beta_{2}=-[\frac{c+\sqrt{c^{2}+4M_{*}\mu_{*}}}{2\mu_{*}}]. \end{array}$$ (60)

The meaningful solution for the velocity field *F*(*y*, *t*) is obtained corresponding to *β*_2_. This solution can be written as

$$\begin{array}{c} F\left(y,t\right)=\text{exp}[-\frac{\left(c+\sqrt{c^{2}+4M_{*}\mu_{*}}\right)}{2\mu_{*}}\left(y+ct\right)], \end{array}$$ (61)

subject to the specific values of arbitrary functions *V*(*t*) and *H*(*y*) as

$$\begin{array}{c} F\left(0,t\right)=V\left(t\right)=\text{exp}(-\frac{c^{2}+c\sqrt{c^{2}+4\mu_{*}M_{*}}}{2\mu_{*}}t)\\ F\left(y,0\right)=H\left(y\right)=\text{exp}(-\frac{c+\sqrt{c^{2}+4\mu_{*}M_{*}}}{2\mu_{*}}y). \end{array}$$ (62)

It can be noticed from the above equation that *V*(*t*) and *H*(*y*) depends on the physical parameters of the flow.

In order to find the numerical solution of Eq (56) for *n* ≠ 1, Eq (56) is first reduces into a system of first order ordinary differential equations

$$\begin{array}{c} f_{3}^{\prime }=q,\;q^{\prime }=\frac{M_{*}f_{3}-cq}{n\mu_{*}q^{n-1}}, \end{array}$$ (63)

$$\begin{array}{c} f_{3}\left(\xi \right)=1,\;at\;\xi =0,\;f_{3}\left(\xi \right)\to 0\;as\;\xi \to \infty . \end{array}$$ (64)

The initial guess is necessary to approximate *q*(*ξ*) at *ξ* = 0. This can be made by using collocation method. The MATLAB bvp4c solver is used to approximate numerical solutions for the *Cu*-water nanofluid.

#### Graphical results and discussion

**Fig 8:**
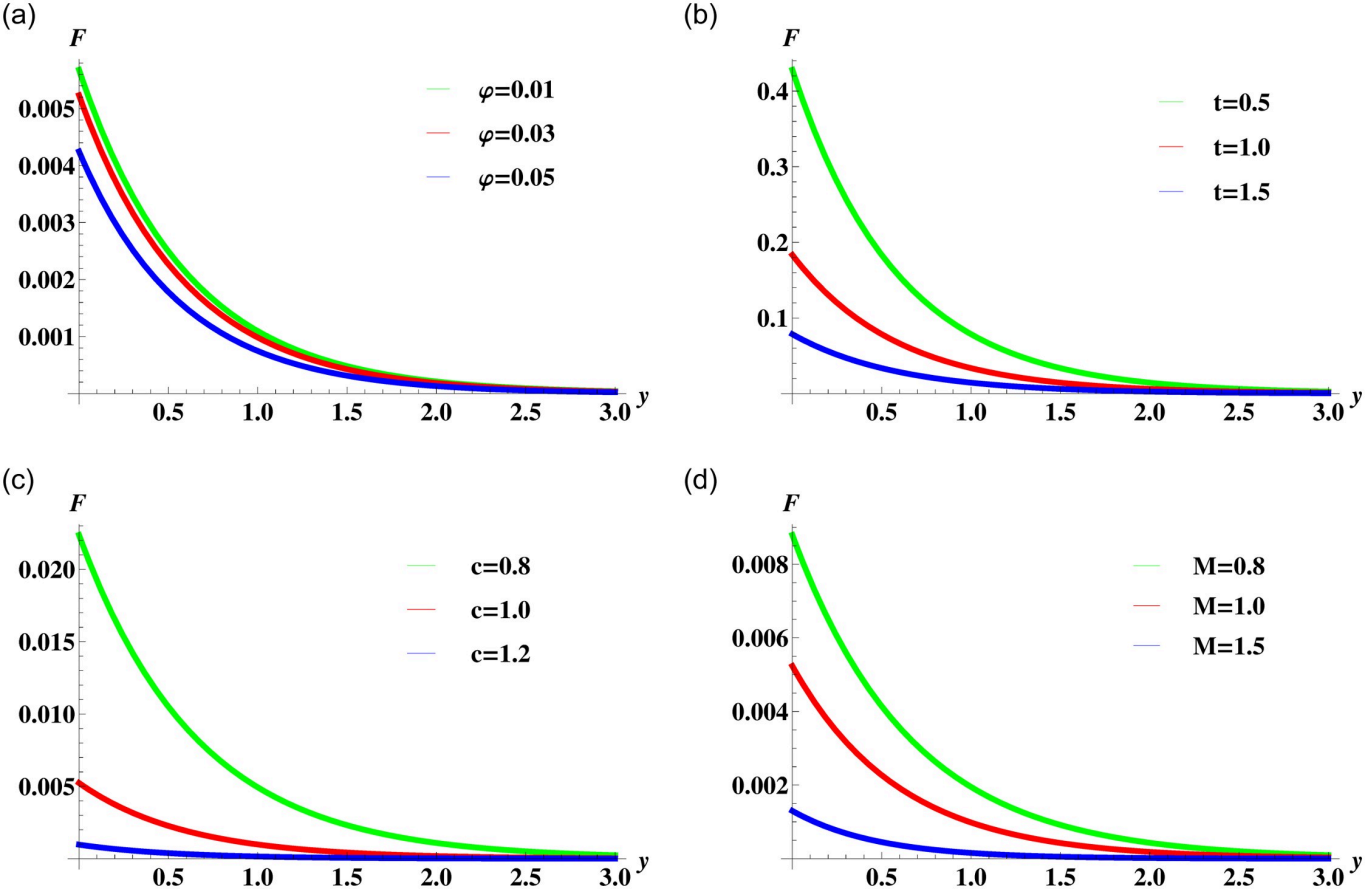
Velocity profile (61) for various values of *φ*, *t*, *c* and *M* when *ρ*_*s*_ = 8933, *ρ*_*f*_ = 997.1 *σ*_*s*_ = 5.5 × 10^−6^ and *σ*_*f*_ = 5.96 × 10^7^ are fixed.

The solution corresponding to a linear combination of time and spatial translation is plotted in Fig 8. The effect of nanoparticles volume concentration, time and magnetic parameters are the same as discussed in previous cases. Here, the effect of the speed of traveling waves on fluid motion is of interest. The parameter *c* is inversely proportional to the fluid velocity. This fact is evident from the curves in Fig 8(*c*).

**Fig 9:**
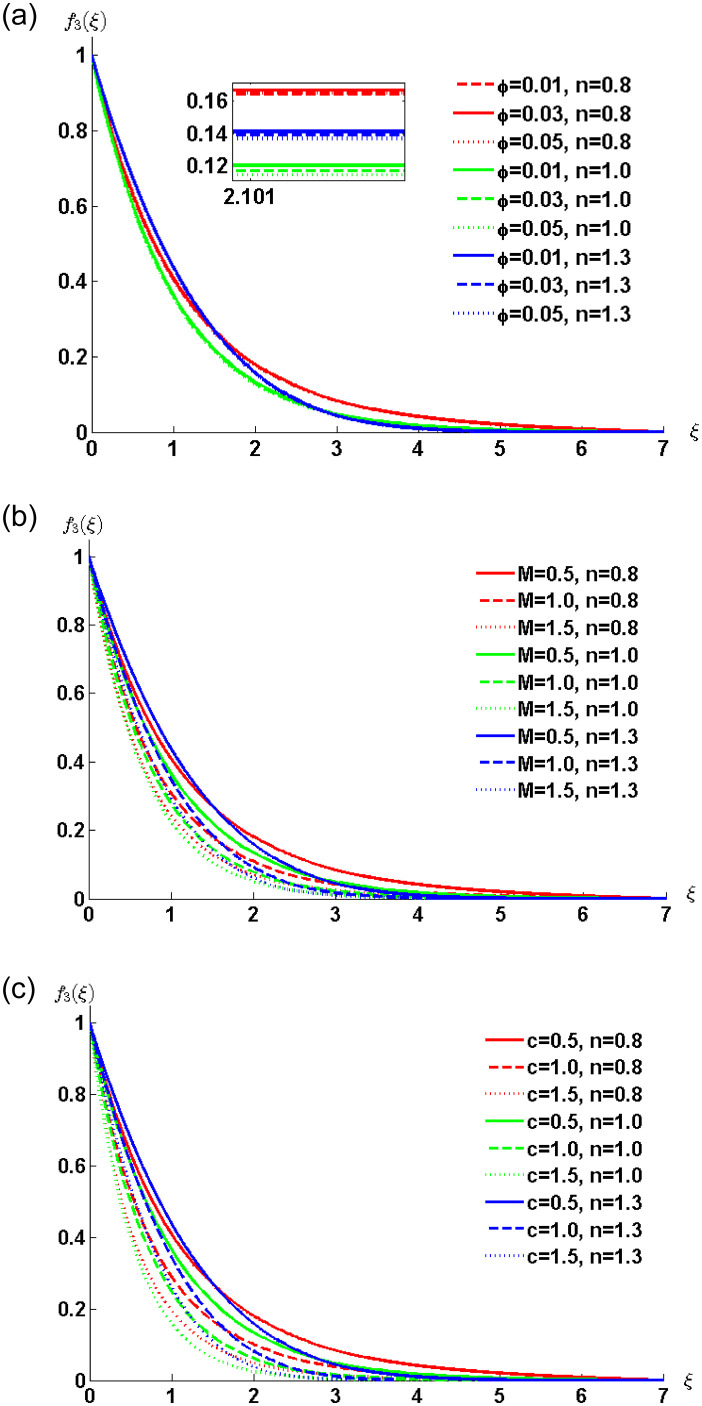
Velocity profile for various values of *φ*, *M* and *c* when *ρ*_*s*_ = 8933, *ρ*_*f*_ = 997.1, *σ*_*s*_ = 5.5 × 10^−6^ and *σ*_*f*_ = 5.96 × 10^7^ are fixed.

The numerical solution computed through MATLAB bvp4c solver for the case of non-Newtonian nanofluid flow model is plotted in the Fig 9. In Fig 9(*a*) the effects of nanoparticles volume concentration is presented with the increasing values of the power-law index. The figure elucidates that, the increase in nanoparticles volume concentration parameter *ϕ* decreases nanofluid velocity within the boundary layer. The thickening of a momentum boundary layer is observed for shear thinning, Newtonian and shear thickening nanofluids. Fig 9(*b*) depicts the behaviour of the magnetic field on the fluid velocity. It can be observed that the increasing values of a magnetic parameter are responsible for the decrease in the fluid velocity. Fig 9(*c*) illustrates the effect of the speed of waves on the velocity of the fluid. The augmentation in wave speed *c* decreases the velocity of nanofluid for all three cases, i.e. pseudo-plastic, Newtonian and dilatant nanofluids.

## Group invariant solution of heat transfer model

Travelling wave solution is the special kind of solution that remains invariant under the linear combination of time and space translation. The solution was discussed previously for the flow equation as well. Considering the combination of operators as Ω_1_ − *c*Ω_2_, with *c* as the constant wave speed (*c* > 0). This combination gives the exact solution as

$$\begin{array}{c} T(y,t)=g(\eta ),\;\text{where}\;\eta =y+ct. \end{array}$$ (65)

Using Eq (65) into Eq (13) gives the second order ordinary differential equation

$$\begin{array}{c} cg^{\prime }\left(\eta \right)-\frac{\left(Nr+\kappa_{*}\right)}{Pr{\left(\rho C_{p}\right)}_{*}}g^{\prime \prime }\left(\eta \right)=0. \end{array}$$ (66)

The above equation admits the exact solution of the form

$$\begin{array}{c} g\left(\eta \right)=\text{exp}(\frac{-c{\left(\rho C_{p}\right)}_{*}Pr}{\left(Nr+\kappa_{*}\right)}\eta ). \end{array}$$ (67)

The boundary conditions (14)–(16) for *g*(*η*) are

$$\begin{array}{c} g(0)=1,\;\;\;\;\;g(\eta )\to 0\;\;\;\;\text{as}\;\;\;\;\;\eta \to \infty . \end{array}$$ (68)

Substituting back Eq (67) into Eq (65) gives

$$\begin{array}{c} T\left(y,t\right)=\text{exp}(\frac{-c{\left(\rho C_{p}\right)}_{*}Pr}{\left(Nr+\kappa_{*}\right)}\left(y-ct\right)), \end{array}$$ (69)

which satisfies the boundary conditions with specific values of *v*(*t*) and *h*(*y*) as

$$\begin{array}{c} T\left(0,t\right)=v\left(t\right)=\text{exp}(\frac{c^{2}{\left(\rho C_{p}\right)}_{*}Pr}{\left(Nr+\kappa_{*}\right)}t),\\ T\left(y,0\right)=h\left(y\right)=\text{exp}(\frac{-c{\left(\rho C_{p}\right)}_{*}Pr}{\left(Nr+\kappa_{*}\right)}y). \end{array}$$ (70)

The above expressions of *v*(*t*) and *h*(*y*) depends on the physical parameters of heat transfer.

### Graphical results and discussion

**Fig 10:**
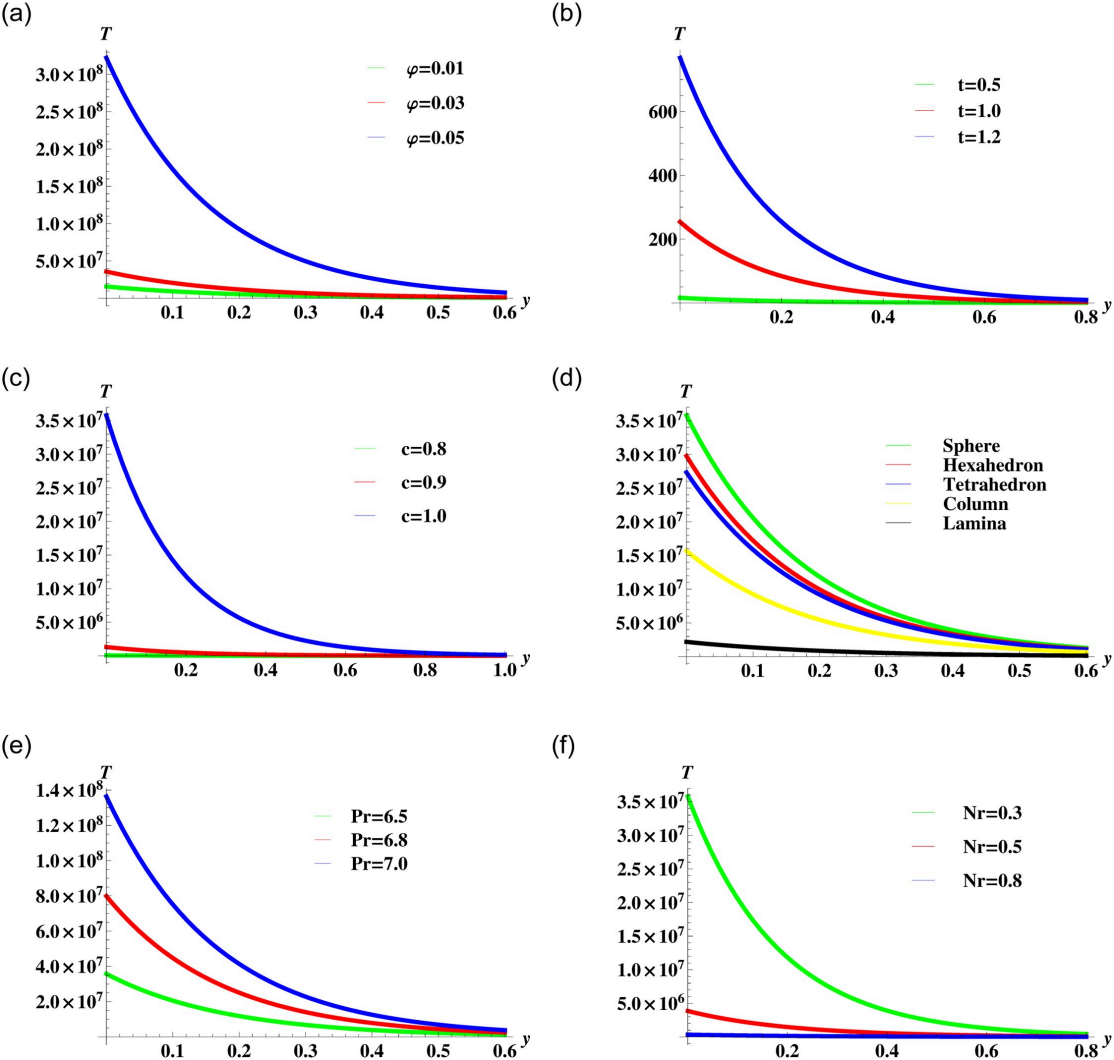
Temperature profile (69) for various values of *φ*, *t*, *c*, *P*_*r*_, *N*_*r*_ and *m* when *ρ*_*s*_ = 8933, *ρ*_*f*_ = 997.1 *σ*_*s*_ = 5.5 × 10^−6^, *σ*_*f*_ = 5.96 × 10^7^ *Cp*_*s*_ = 385, *Cp*_*f*_ = 4197, *κ*_*s*_ = 400, and *κ*_*f*_ = 0.613 are fixed.

The heat transfer analysis for different parameters that affect the temperature of the fluid corresponding to the solution (69) are depicted in Fig 10(*a*)–10(*f*). The effect of nanoparticles volume concentration is shown in Fig 10(*a*). It is clear from the figure that the increasing values of *φ* causes an increase in the temperature within the boundary layer. This happens due to the increase in the overall thermal conductivity of the nanofluid. The time-lapse effects on the temperature of the fluid are shown in Fig 10(*b*). The curves present an increase in temperature with time. The effect of traveling waves speed *c* are plotted in Fig 10(*c*). The maximum temperature of the fluid is near the boundary but when the waves propagates away from the boundary the temperature falls. The effect of the nanoparticles shape parameter is also responsible for heat transfer rate. These effects are visualized graphically in Fig 10(*d*). The graphical view reveals that the spherical shape has the highest temperature. The spherical-shaped particles tend to drag more heat from the boundary because of their maximum surface area. The Prandtl number is the ratio of momentum to thermal diffusivity so it is obvious when there is a high thermal conductivity the temperature will rise. This fact is evident from the curves in Fig 10(*e*). The presence of thermal radiation reduces the fluid temperature and decreases the thickness of the thermal boundary layer as depicted in Fig 10(*f*).

## Conclusion

The present model aims to find the exact (closed-form) solution of the unsteady unidirectional flow and heat transfer model of power-law nanofluid. The fluid is triggered by the motion of plate. The uniform magnetic field is applied in the flow direction. The thermal motion of the particles causes the radiation with in the fluid. The governing equations for the flow and heat transfer model are solved using the Lie symmetry approach. The method leads to reductions and closed-form solution for the different infinitesimal generators. The behaviour for shear thinning, Newtonian and shear thickening fluids are discussed in each case for various physical parameters. The graphs given in discussion section are separately described in all aspects. The following deductions are made after the results:

- The increasing volume concentration of nanoparticles causes a decrease in velocity and increase in temperature.
- The velocity of the fluid is decreasing with the increasing strength of the magnetic field.
- The fluid motion becomes slow and the temperature rises as time goes on.
- The shear thickening fluid has a thick momentum boundary layer as compared to Newtonian and shear thinning fluid.
- The increasing wave speed reduces the fluid motion and increases temperature.
- The higher thermal conductivity rises the fluid temperature.
- The increase in thermal radiation causes to lower the temperature of the fluid.
- The spherical shaped nanoparticles are more likely to drag additional heat within the fluid.

In the light of the above findings, the behaviour for different physical parameters admits the same performance that fulfils the established facts. The methodology used in this study is more systematic and general. This approach is very helpful in finding the exact solution especially related to non-linear partial differential equations. The generalized compatibility approach aided this technique for the closed-form solutions. There is enormous space available in the literature to use this technique in the fields like general relativity, aerodynamics and wave and solid mechanics.
